# Ascending aorta backward flow parameters estimated from phase-contrast cardiovascular magnetic resonance: new indices of vascular aging

**DOI:** 10.1186/1532-429X-15-S1-P230

**Published:** 2013-01-30

**Authors:** Zoubir M Bensalah, Emilie Bollache, Nadjia Kachenoura, Alban Redheuil, Elie Mousseaux

**Affiliations:** 1 Radiology, Hospital Ambroise Pare, Paris, France; 2 Cardiovascular Radiology, Hospital George Pompidou, Paris, France; 3 Laboratoire d'imagerie Fonctionnelle, INSERM, Paris, France

## Background

Aging, a major cardiovascular risk factor, is associated with arterial stiffness. PC-CMR blood flow studies can assess the presence of both Backward flow (BF) and forward flow (FF) in the aorta. We hypothesized that quantitative parameters derived from could be relevant markers of arterial aging and stiffness.

## Methods

One hundred healthy subjects (50 women, mean age: 40±15 years (19-79 years) were studied. Aortic stiffness (aortic arch pulse wave velocity PWVAO, AA distensibility), aortic arch geometry (AA diameter and arch length) and parameters related to AA global backward and forward flows (volume, flow rate peak, and onset time) were estimated from CMR. Carotid-femoral pulse wave velocity (PWVCF), as well as reflection parameters such as carotid augmentation index (AIx) and time to return of the reflected pressure wave (Ti) were assessed by tonometry.

## Results

BF volume and flow rate peak increased and time to onset of BF was shortened as age increased (P<0.0001), while global aortic flow and FF parameters were unchanged. The BF to FF peaks ratio was the parameter most strongly related to both age and arterial stiffness indices. In multivariate analysis, the independent associates of BF were age, AA diameter, aortic arch length and AIx.

## Conclusions

AA BF parameters estimated in PC-CMR were related to age, aortic geometry and to arterial wave reflections and could be relevant markers of subclinical vascular aging.

## Funding

Nothing

